# HERG potassium channels are more frequently expressed in human endometrial cancer as compared to non-cancerous endometrium

**DOI:** 10.1054/bjoc.2000.1497

**Published:** 2000-12

**Authors:** A Cherubini, G L Taddei, O Crociani, M Paglierani, A M Buccoliero, L Fontana, I Noci, P Borri, E Borrani, M Giachi, A Becchetti, B Rosati, E Wanke, M Olivotto, A Arcangeli

**Affiliations:** Department of Experimental Pathology and Oncology, University of Firenze, Viale G.B. Morgagni 50, Firenze, 50134, Italy; Department of Human Pathology and Oncology, University of Firenze, Viale G.B. Morgagni 85, Firenze, 50134; Department of Gynecology and Human Reproduction, University of Firenze, Viale G.B. Morgagni 85, Firenze, 50134; Department of Biotechnology and Biosciences, University of Milano Bicocca, Piazza della Scienza 2, Milano, 20126, Italy

**Keywords:** HERG, endometrial cancer, endometrial hyperplasia, tumour markers, K^+^channels

## Abstract

HERG K^+^channels, besides contributing to regulate cardiac and neuronal excitability, are preferentially expressed in tumour cell lines of different histogenesis, where their role in the development and maintenance of the neoplastic phenotype is under study. We show here that both *herg* gene and HERG protein are expressed with high frequency in primary human endometrial cancers, as compared to normal and hyperplastic endometrium. RT-PCR and immunohistochemistry, using specific anti-HERG antibodies developed in our laboratory, were applied to tissue specimens obtained from 18 endometrial cancers and 11 non-cancerous endometrial tissues. *herg* RNA and HERG protein are expressed in 67% and 82%, respectively, of cancerous, while in only 18% of non-cancerous tissues. In particular, no expression was found in endometrial hyperplasia. Moreover, electrophysiological experiments confirmed the presence of functioning HERG channels on the plasma membrane of tumour cells. On the whole, these data are the first demonstration of the presence of HERG channels in primary human neoplasias, and could candidate HERG as a potential tool capable of marking cancerous versus hyperplastic endometrial growth. © 2000 Cancer Research Campaign http://www.bjcancer.com


*British Journal of Cancer* (2000) **83**(12), 1722–1729
© 2000 Cancer Research Campaign
doi: 10.1054/ bjoc.2000.1497, available online at http://www.idealibrary.com on IDEAL®

http://www.bjcancer.com

### HERG potassium channels are more frequently expressed in human endometrial cancer as compared to non-cancerous endometrium

**A Cherubini[1], GL Taddei[2], O Crociani[1], M Paglierani[2], AM Buccoliero[2], L Fontana[1], I Noci[3], P Borri[3], E Borrani[3], M Giachi[3], A Becchetti[4], B Rosati[4], E Wanke[4], M Olivotto[1] and A Arcangeli[1]**

[1]Department of Experimental Pathology and Oncology, University of Firenze, Viale G.B. Morgagni, 50, 50134 Firenze, Italy; [2]Department of Human Pathology and Oncology, University of Firenze, Viale G.B. Morgagni, 85, 50134 Firenze; [3]Department of Gynecology and Human Reproduction, University of Firenze, Viale G.B. Morgagni, 85, 50134 Firenze; [4]Department of Biotechnology and Biosciences, University of Milano Bicocca, Piazza della Scienza, 2, 20126 Milano, Italy

**Summary** HERG $K^+$ channels, besides contributing to regulate cardiac and neuronal excitability, are preferentially expressed in tumour cell lines of different histogenesis, where their role in the development and maintenance of the neoplastic phenotype is under study. We show here that both *herg* gene and HERG protein are expressed with high frequency in primary human endometrial cancers, as compared to normal and hyperplastic endometrium. RT-PCR and immunohistochemistry, using specific anti-HERG antibodies developed in our laboratory, were applied to tissue specimens obtained from 18 endometrial cancers and 11 non-cancerous endometrial tissues. *herg* RNA and HERG protein are expressed in 67% and 82%, respectively, of cancerous, while in only 18% of non-cancerous tissues. In particular, no expression was found in endometrial hyperplasia. Moreover, electrophysiological experiments confirmed the presence of functioning HERG channels on the plasma membrane of tumour cells. On the whole, these data are the first demonstration of the presence of HERG channels in primary human neoplasias, and could candidate HERG as a potential tool capable of marking cancerous versus hyperplastic endometrial growth. © 2000 Cancer Research Campaign http://www.bjcancer.com

**Keywords:** HERG; endometrial cancer; endometrial hyperplasia; tumour markers, $K^+$ channels

*herg* (*human eag related gene*) encodes a particular type of $K^+$ channel (HERG) belonging to an evolutionary conserved multigene family of voltage-activated, outward rectifying $K^+$ channels, the *eag* (*ether a-gò-gò*) family (Warmke and Ganetzky, 1994). The corresponding current ($I_{HERG}$) is characterized by inward rectification properties, and its role is well known in the heart, where it contributes to the repolarization of the cardiac action potential (Sanguinetti et al, 1995), and is being discovered in neurons, where it appears to regulate spike-frequency adaptation (Chiesa et al, 1997). We first demonstrated that *herg* and its related current are preferentially expressed in neoplastic cell lines of different histogenesis (Arcangeli et al, 1993, 1995; Faravelli et al, 1996; Bianchi et al, 1998); in tumour cells, $I_{HERG}$ is responsible for maintaining substantially depolarized resting potentials, a recurrent feature in cancer cells (Binggeli and Weinstein, 1986; Olivotto et al, 1996). Following this first discovery, another member of the family, the EAG channel, has been found to be preferentially expressed in tumour cell lines, and to confer oncogenic properties to transfected cells (Pardo et al, 1999). On the whole, a functional role in oncogenesis can be tentatively attributed to genes belonging to the *eag* family of $K^+$ channels.

On these bases we have undertaken a study to evaluate *herg* expression in primary human tumours, to be compared with the correspondent normal or hyperplastic tissues. The endometrial cancer (EC) was chosen for this purpose: in fact, besides being nowadays the commonest invasive malignancy of the female genital tract (Burton and Wells, 1998), the molecular pathogenesis of EC is still largely unknown (Berchuck and Boyd, 1995), despite the numerous genomic alterations so far reported, such as abnormalities of ploidy (Milatovich, 1990), instability of microsatellite sequences (Risinger et al, 1993; Burks, 1994), altered expression of various genes (reviewed by Burton and Wells, 1998; Baker, 1996). In this context, special attention has been given to point mutations of the p53 tumour suppressor gene, leading to an overexpression of mutant p53 protein (Porter et al, 1992; Koheler et al, 1993), expression of the oncogene bcl-2 (Taskin et al, 1997) and of oestrogen and progesterone receptor (ER and PR) (Creasman, 1993). Despite these studies, no molecular marker is nowadays as valuable in determining prognosis as conventional histopathological parameters, in particular the histopathological grading. Thus, the study of the molecular events underlying EC tumorigenesis, as well as the determination of new, specific, molecular markers characterizing EC are deeply encouraged, to help the pathologist in the diagnostic and prognostic evaluation of EC.

We present here a study showing that *herg* and HERG protein are much more frequently expressed in EC as compared to non-cancerous (normal and/or hyperplastic) endometrium (NCE); RT-PCR analysis has been performed in parallel with immunohistochemical assays, and patch clamp recordings: all techniques lead to the same result that *herg* RNA and HERG protein expression marks a high percentage of EC, as compared to NCE.

*Received 25 May 2000*
*Revised 31 July 2000*
*Accepted 10 August 2000*

*Correspondence to:* A Arcangeli

### MATERIALS AND METHODS

### Tissue collection

Surgical specimens were obtained from 28 women undergoing hysterectomy for endometrial cancer (17 cases), for leiomyoma uteri (5 cases), for prolapsus uteri (2 cases), for ovarian cystic disease (2 cases) and for intractable methrorrhagia (2 cases). One histological specimen of EC was obtained from endometrial biopsy. Samples used for RT-PCR analysis were obtained from the hysterectomy specimens after a gentle and superficial scraping of the endometrial tissue, using a sterile curette, from fresh, non-fixed uterine body, longitudinally cut. Endometrial tissue was in any case immediately frozen; several histological frozen sections obtained from a portion of the endometrial tissue were immediately examined in order to verify the absence of myometrium; the latter could in fact lead to ambiguous results (see Results); for this reason, hypo/atrophic specimens, as well as all samples that resulted contaminated, were excluded from our study and only pure endometrial tissue was utilized for RT-PCR analysis. Histological and immunohistochemical evaluations were performed on uterine tissues routinely sampled and processed, from either hysterectomy specimens and endometrial biopsy tissue.

Gynaecological pathologists (GLT, AMB) using standard criteria assessed the histological diagnosis. In 18 women, whose mean age was 62 years (range 45–85 years), the diagnosis was an endometrial cancer: among these, 11 cases were usual endometrioid adenocarcinoma, 3 were adenocarcinoma with squamous metaplasia, 2 cases were serous-papillary carcinoma, 1 secretory adenocarcinoma and 1 case was mesonephroid or clear cell adenocarcinoma. Four patients had good differentiation (G1), 7 had intermediate (G2) and 5 poor differentiation (G3). For 2 serous-papillary carcinoma grading was not performed, although they can be compared with poorly differentiated, G3. Histological diagnosis in the remaining 11 women was proliferative endometrium (1 case), secretive endometrium (4 cases), and simple endometrial hyperplasia without atypia (6 cases). The median age was 47 years in women with proliferative or secretive endometrium (range 42–54), and 56 years (range 49–75) in simple hyperplasia.

### Histological examinations and immunohistochemistry

The histological study on endometrial samples was performed at the Department of Pathology, University of Florence, Italy. Endometrial specimens were routinely fixed in buffered formalin and embedded in paraffin. The pathologists evaluated the endometrial histology on sections stained with haematoxylin and eosin. Several consecutive sections were used for immunohistochemical study. Once the sections had been mounted on electrostatic slides and air-dried overnight at 37˚C, they were deparaffinized through xylene and rehydrated through a graded alcohol series. The endogenous peroxidase activity was blocked by immersing the specimens in a solution of 0.5% $H_2O_2$ in distilled water. To recover antigenicity, slides were placed in 10 mM citrate buffer pH 6.0 and heated in a household microwave oven at 300 W for 40 min. After microwave processing, the sections were allowed to cool down for 20 min at room temperature, washed with phosphate buffered saline solution (PBS, pH 7.4) and treated with normal horse serum (LAB VISION Corporation Fremont, CA, USA) to reduce non-specific antibody binding.

Several different primary antibodies were used: monoclonal antibody 1D5 raised against human oestrogen receptor (1:30 dilution, Bio-Genex, San Ramon, CA, USA); monoclonal antibody 1A6 raised against human progesterone receptor (1:50 dilution, Bio-Genex); a murine monoclonal antibody IgG2 antibody DO-7 raised against human p53 (1:40 dilution, DAKO A/S, Denmark), which reacts with wild type and mutant p53 protein and a murine monoclonal (clone 124, IgG1) anti-human bcl-2 antibody (1:20 dilution, DAKO). After washing with PBS, sections were incubated with biotinylated goat anti polyvalent antibody (LAB VISION) and then with streptavidin-biotin-peroxidase complex reagent (LAB VISION). After extensive washing with PBS, slides were treated with 3,3′-diaminobenzidine-hydrogen peroxide (Bio-Genex), as the final indicator, and counterstained lightly with Mayer’s haematoxylin. Negative control experiments were performed by replacing the primary antibodies with non-immune mouse serum at an equivalent protein concentration.

Histological sections were also treated with polyclonal anti-HERG antibody (1:1000 dilution, obtained in Dr Arcangeli’s laboratory, see below), using the previously described standard method of immunoperoxidase staining without microwave. Positive control experiments of anti-HERG antibodies were performed on sections relative to a human heart (explanted from a patient suffering from cardiomyopathy), as well as on SY5Y neuroblastoma cell line, cultured in DMEM + 10% fetal calf serum (FCS) as previously described (Arcangeli et al, 1995). In this case, cells were cultured on a glass slide for 48 hours, then washed in PBS and fixed in absolute ethanol for 10 min, then processed as above. Negative control experiments were performed treating the histological sections with a non-immune rabbit serum (DAKO) instead of the primary anti-HERG serum. Some sections were also immunolabelled using a polyclonal anti-HERG C-terminus antibody, kindly gifted by Dr JM Nerbonne (University of Washington, St Louis, USA). The nuclear positivity of oestrogen and progesterone receptors, p53, and cytoplasmic positivity of bcl-2, was evaluated by estimating the fraction of positive glandular cells on the total number of the glandular cells, in 10 separate fields at × 40 HPF. According to Taskin et al (1997), the immunostaining was scored with 0 (0–5%), 1 (6–10%), 2 (11–50%), 3 (>50%). The cytoplasmic, or rarely nuclear, HERG immunoreaction was qualitatively scored with + when present, with – when no immunostaining was recognizable. Sometimes a focal positivity to anti-HERG antibodies was observed.

Statistical analysis was performed using the Chi-square, as well as the Fisher’s test.

### Production of anti-HERG antibodies

Anti-HERG antibodies were produced by immunizing rabbits with a fusion protein, representing the highly conserved N-terminal amino acids of the HERG sequence from 1 to 135 (Li et al, 1997). Expression of the glutathione S-transferase (GST) fusion protein was carried out using pGEX-4T-2 vector (Pharmacia). The *herg* coding sequence was obtained by amplifying the *herg1* cDNA in SP6 vector (kindly gifted by Dr Keating) using the following primers:

sense:
GGGTCGACAATGCCGGTGCGGAGG
antisense:
CAGGCGGCCGCCTACTTCTCCATCACCACC

© 2000 Cancer Research Campaign

A SalI and NotI cutting sequence was inserted in the *herg* sequence, so that the PCR product could be ligated in frame in the pGEX-4T-2 vector; the insert was sequenced to exclude sequence errors due to the Taq amplification; the N-terminus *herg*-containing *E. coli* (DH5α) colonies were amplified and the GST fusion protein purified according to the manufacturer's protocol; no cut was applied to eliminate the GST peptide from the fusion protein. 500 μg of fusion protein were injected subcutaneously in adult rabbits along with complete Freund's adjuvant; two subsequent injections of 250 μg each were applied, and the level of specific antibodies was evaluated by an ELISA test. The polyclonal antiserum was then aspirated, aliquoted and stored at –80˚C. The antiserum was tested for its anti-HERG activity in immunoblot experiments on membrane extracts obtained from HERG endowed tissues (see below), and comparing its immunoreactivity with Dr Nerbonne's anti-HERG antibody.

### Immunoblot

Immunoblot with anti-HERG antibodies was performed on a crude membrane fraction prepared from a mouse brain, essentially according to Pond et al (2000). Membrane proteins were separated by SDS polyacrylamide gel electrophoresis (SDS-PAGE) and transferred to a nitrocellulose sheet. After transfer, membranes were blocked for 4 h at room temperature with PBS+Tween-20 0.1% containing 5% BSA (T-PBS-BSA) and then incubated overnight at 4˚C with Dr Nerbonne's rabbit anti-HERG polyclonal antibody diluted 1:500 in T-PBS-BSA, or with the raw serum containing polyclonal anti-HERG antibody obtained in Dr Arcangeli's laboratory (see above) diluted 1:5000 in T-PBS-BSA. Membranes were then washed 3 times with T-PBS and incubated with anti-rabbit peroxidase-conjugate secondary antibodies (Sigma; diluited 1:10 000 in T-PBS-BSA) for 1 h at room temperature. After 3 washes with T-PBS, the immunoreactivity was determined by a chemiluminescent reaction (ECL) (Amersham).

### Reverse transcription (RT) and polymerase chain reaction (PCR) amplification

Frozen tissues were homogenized by a guanidinium thiocyanate solution, and total RNA was extracted using 2 M sodium acetate pH 4, acid phenol, and chloroform-isoamyl alcohol (49:1). The aqueous phase, obtained after centrifugation at 15 000 ***g*** for 15 min, was precipitated overnight at –20˚C with ethanol. The pellet was washed twice with 70% ethanol and dissolved in 1 mM EDTA, 10 mM NaCl, 10 mM Tris-HCl, pH 8.0. RNA purity and integrity was checked by running an aliquot on a 1% agarose gel (Maniatis et al, 1989). 1–2 μg were retrotranscribed in a 20 μl reaction by MuLV (Murine Leukaemia Virus Reverse Transcriptase) (Perkin Elmer) 2.5 U $\mu l^{-1}$, 5 mM $MgCl_2$, 1 mM d(NTP)s, 1X PCR buffer, RNAse Inhibitor 1 U $\mu l^{-1}$ and 2.5 μM random hexamer primers, for 30 min at 42˚C. 5 μl of cDNA were then amplified by the polymerase chain reaction in a 50 μl reaction containing AmpliTAQ Gold Polymerase 5 U $\mu l^{-1}$ (Perkin Elmer), 2.5 mM $MgCl_2$, 200 μM d(NTP)s, 1X PCR buffer. The sequence of ologinucleotide primers was as follows:

Primer sense:
5′-TCCAGCGGCTGTACTCGGGC -3′
Primer antisense:
5′-TGGACCAGAAGTGGTCGGAGAACTC -3′

These primers comprise a sequence between nucleotide 2171 to nucleotide 2746 of the *herg* sequence (accession number HU04270). We carried out 43 cycles of amplification after 10 min of enzyme activation at 94˚C: denaturation at 94˚C for 1.5 min, annealing at 65˚C for 3 min, extension at 72˚C for 1.5 min DNA. Products were run on a 2% agarose gel using a molecular weight marker, the 100 bp DNA ladder (New England Biolabs) and bands were visualized by ethidium bromide staining on a UV transilluminator. Control amplifications were performed either adding not retrotranscribed RNA or no-DNA, no-RNA in the PCR tube, but any aspecific band was never observed. This latter control was performed every time.

cDNA samples were checked for integrity by PCR detection of human *gapdh* using AmpliTAQ Polymerase 5 U $\mu l^{-1}$ (Perkin Elmer), 2 mM $MgCl_2$, 200 μM d(NTP)s, 1X PCR buffer and the following primers, which comprise a sequence between nucleotide 457 to nucleotide 595 of *gapdh* gene:

Primer sense:
5′-AACAGCCTCAAGATCATCAGCAA-3′
Primer antisense:
5′-CAGTCTGGGTGGCAGTGAT-3′

PCR conditions were as follows: denaturation at 94˚C for 1 min, annealing at 60˚C for 1 min, extention at 72˚C for 1 min, for 35 cycles.

A sample of human blood was analysed by RT-PCR amplification of *herg* gene, but any product was never observed, confirming that blood does not interfere with *herg* amplification in tissue samples. The amount of blood used for RNA extraction and subsequent RT-PCR analysis was 50 μl, considering it the maximum aliquot of possible contamination of tissue samples, which were never more than 500 mg in size (10% blood/tissue).

### Sequencing of PCR products

PCR product obtained after *herg* amplification in one sample of adenocarcinoma was submitted to automatic sequencing (MWG-Biotech GmbH) to confirm the homology between the 575 bp PCR product and *herg* gene.

### Patch-clamp recordings

Patch-clamp recordings were performed on primary cell cultures obtained from endometrial adenocarcinoma samples, essentially according to Chatzaki et al (1994). Briefly, hysterectomy specimens were first trimmed and minced, then dissociated through a first digestion with collagenase II (Roche, 0.05 mg $ml^{-1}$ final concentration) at 37˚C for 2 hours, and a second treatment with trypsin/EDTA solution (0.5 g $ml^{-1}$ trypsin, 0.2 g $ml^{-1}$ tetrasodium EDTA, Sigma) for 10 min at room temperature with constant agitation. Cells were then washed in complete medium (Waymouth's medium supplemented with 10% fetal calf serum (FCS) (Characterized, Hyclone) and 1% penicillin-streptomycin/fungizone mixture (Hyclone)), resuspended in the same medium and seeded into 35 mm Petri dishes (Costar). After 24 hours of incubation, cell cultures were washed twice with PBS, and fresh medium was added. The culture medium was then replaced every two days.

Patch-clamp recordings were performed at room temperature with an amplifier Axopatch 1-D (Axon Instruments, Foster City, CA), replacing the Petri dish every 30 min. The whole cell configuration

 *© 2000 Cancer Research Campaign*

of the patch clamp technique (Hamill et al, 1981) was employed, using pipettes (borosilicate glass; Hilgenberg, Germany) whose resistance was in the range of 3–5 MΩ. Extracellular solutions were delivered through a 9-hole (0.6 mm), remote-controlled linear positioner placed near the cell under study. The standard extracellular solution contained (mM): NaCl 130, KCl 5, $CaCl_2$ 2, Hepes-NaOH 10, glucose 5, pH 7.4. The extracellular solution with high $K^+$ contained (mM): NaCl 95, KCl 40, $CaCl_2$ 2, Hepes-NaOH 10, glucose 5, pH 7.4. The standard pipette solution at $[Ca^{2+}] = 10^{-7}$ M contained (mM): $K^+$ aspartate 130, NaCl 10, $MgCl_2$ 2, $CaCl_2$ 2, EGTA-KOH 10, Hepes-KOH 10, pH 7.4. Gigaseal resistances were in the range of 3–20 GΩ. Whole cell currents were filtered at 5 kHz. For precise measurement of the gating parameters of the inward rectifier channels, we carefully compensated pipette and cell capacitance and the series resistance before each voltage-clamp protocol run. Input resistance of the cells was in the range of 2–6 GΩ. The antiarrhythmic drug Way 123,398, kindly gifted by Dr W Spinelli (Wyeth-Ayerst Research, Princeton, NJ), was used at a 1 μM concentration, a condition proved to block HERG currents in cancer cells (Faravelli et al, 1996). For data acquisition and analysis, the pClamp hardware and software (Axon Instruments) and Origin (Microcal Software, Northampton, MA) were routinely used.

### RESULTS

To study the presence of HERG in primary human tumours, we analysed the first available specimens belonging to cancerous, hyperplastic or normal endometria, evaluating the expression of *herg* RNA by RT-PCR, amplifying a 575 bp product (Figure 1). Taking as a control the expression of *herg* in the human neuroblastoma cell line SY5Y (lane 1), the *herg* RNA resulted to be expressed in 2 typical human EC (lanes 2 and 3) but not in normal (lane 4) as well as hyperplastic human endometrium (lane 5). The PCR amplification band relative to one of the tested adenocarcinomas, was purified and submitted to sequencing, resulting identical to the reported sequence of human *herg* (Warmke and Ganetzky, 1994). Other uterine tissues were examined, and a *herg* PCR band was found in normal myometrium (lane 6); this finding led us to exclude from our study all the samples which resulted contaminated by myometrium at the histological analysis and all the hypo/atrophic endometrial samples (see Materials and Methods). On the other hand, a sample of blood, which could possibly contaminate endometrial samples, gave negative results (lane 7). Negative controls were also performed on samples containing not retrotranscribed RNA (lane 8), and no c-DNA no RNA (lane 9). In Figure 1 results are also reported, referring to a RT-PCR amplification of the human *gapdh* gene, taken as an indication of the good quality of the cDNA obtained by retrotranscription. As shown in the figure, the *gapdh* gene resulted positive in all the samples tested, except in the negative controls (lanes 8 and 9, see legend to Figure 1).

On the whole, data reported in Figure 1 demonstrated that the *herg* RNA can be easily detected in human tissues by a simple RT-PCR analysis, and appears to be more frequently expressed in human neoplastic tissues as compared to non-cancerous endometrium. This indication needed to be deepened and confirmed by exploring the presence of HERG protein in EC. Polyclonal anti-HERG antibodies were developed for this purpose (see Materials and Methods). The immunoreactivity of these antibodies was first tested in immunoblot experiments performed on membrane extracts obtained from mouse brain, a tissue highly expressing HERG protein. The immunoreactivity of these anti-HERG antibodies were compared with that displayed by another widely used antibody developed in Dr Nerbonne's laboratory (Pond et al, 2000). Figure 2 shows the results of this experiment: two HERG protein bands can be recognized in the mouse brain, whose molecular weight is about 205 and 165 kDa, either with Dr Nerbonne's antibody (lane A), and with the antibody developed in our laboratory (lane B), according to what was previously reported (Pond et al, 2000).

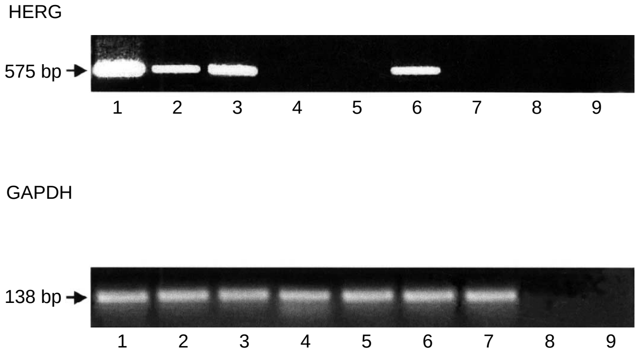

**Figure 1** *herg* RNA expression in normal and tumour tissues. RT-PCR experiments were performed on SY5Y human neuroblastoma cell line (positive control, lane 1), EC (lanes 2–3), endometrium (lane 4), endometrial hyperplasia (lane 5), myometrium (lane 6), blood (lane 7), not retrotranscribed RNA (negative control, lane 8), and no RNA/no cDNA samples (negative control, lane 9), with *herg* and *gapdh* primers (see Material and Methods).

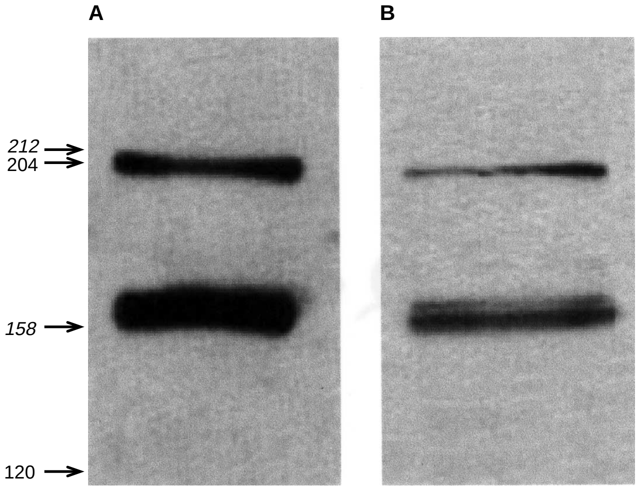

**Figure 2** Western blot analysis of the HERG protein in mouse brain. Mouse brain membranes were prepared, separated on SDS-PAGE and transferred on nitrocellulose sheets as described in Materials and Methods. Membranes were immunolabelled with a polyclonal anti-HERG C-terminus antibody produced in Dr Nerbonne's laboratory (diluted 1:5000, lane A) or with the anti-HERG N-terminus antibody produced in Dr Arcangeli's laboratory (diluted 1:500, lane B). The bands revealed by both antibodies have a molecular weight of 205 and 165 kDa, according to what was reported by Pond et al (2000). Molecular weight standards: italic: New England Biolabs; roman: Biorad.

Therefore, the antibody was tested on the adenocarcinomatous and non-neoplastic tissues previously scored by RT-PCR. SY5Y neuroblastoma cells and cardiac myocytes were chosen for positive control in these experiments. Negative control experiments were also performed with non-immune anti rabbit serum (see Materials and Methods). As shown in Figure 3 (panel A), SY5Y

© 2000 Cancer Research Campaign

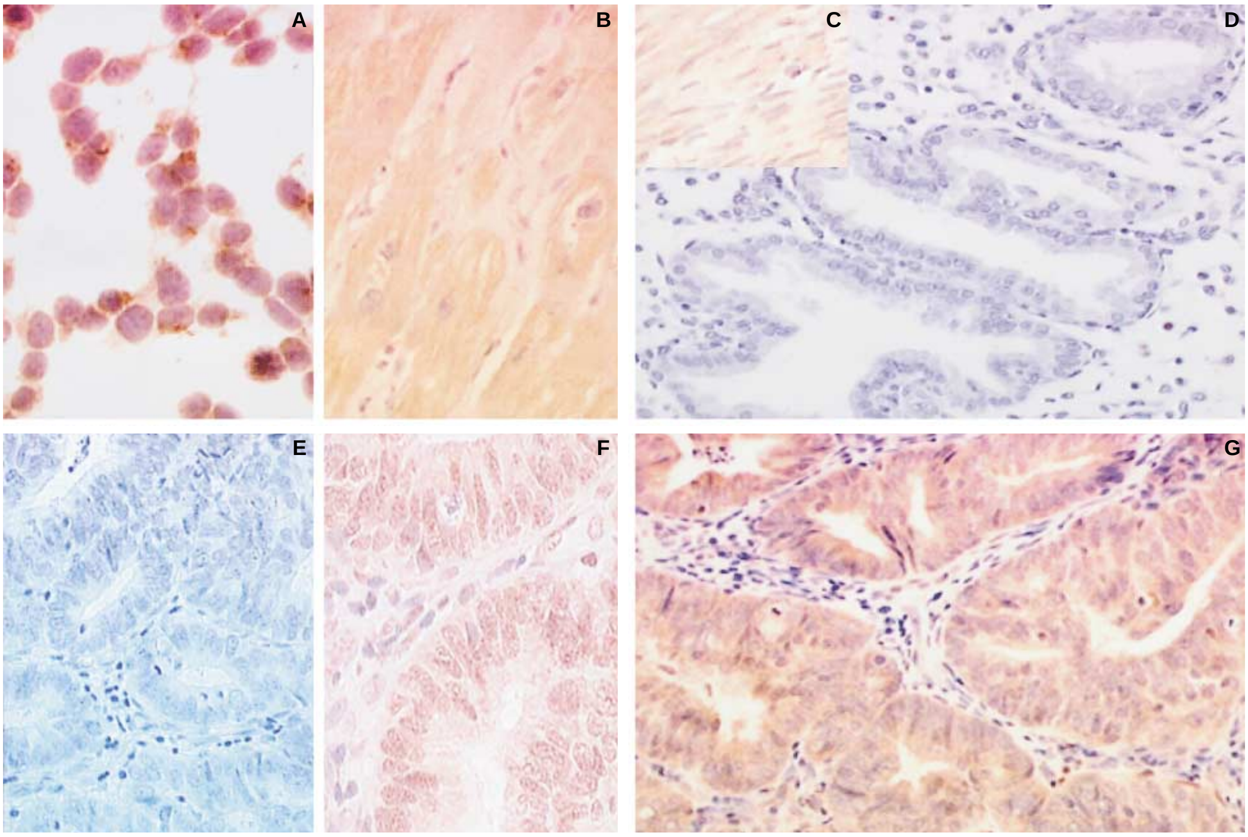

**Figure 3** Immunohistochemical detection of HERG protein. Anti-HERG N-terminus antibodies, developed as reported in Materials and Methods, were used on cells cultured in vitro or on paraffin embedded sections, and revealed by immunoperoxidase staining. A clear cytoplasmic immunostaining is evident in SY5Y human neuroblastoma cell line (**A**), myocardic cells (**B**), glandular adenocarcinomatous cells (**G**) and myometrial cells (inset, **C**). Stromal cells (in **B** and **C**) and glandular cells in a normal secretive endometrium (**D**), as well as adenocarcinoma cells probed with a non-immune rabbit serum (negative control) (**E**) are completely negative. In **F** is reported an adenocarcinomatous sample probed with Dr Nerbonne's anti-HERG antibody. Magnification was × 400 HPF in **A**, × 200 HPF in **B–F**.

cells were positive to anti-HERG antibodies, revealing a prevalent, diffuse cytoplasmic immunostaining, and only rarely, positive nuclei; cardiac muscle cells resulted also positive, while the stromal tissue between muscle fibres was unstained (Figure 3B). On the other hand, the immunohistochemical picture of a normal endometrium turned out to be totally negative for the presence of the HERG protein (Figure 3D). This result appears to be also strengthened by the fact that the muscle cells of myometrium, present in the same section, appeared to be strongly positive (Figure 3C). Adenocarcinomatous tissue (Figure 3G) revealed a good positivity to anti-HERG antibodies, being however negative to a non-immune rabbit serum (Figure 3E); the positivity was here again preferentially localized to the cytoplasm of glandular cells, while the stroma was totally negative. Here again, a control with Dr Nerbonne's antibody is reported, showing a substantially similar pattern of immunoreactivity (Figure 3F), with a slightly higher immunolabelling of adenocarcinomatous cell nuclei. Altogether, data presented in Figure 3 clearly indicate that anti-HERG antibodies can be useful to detect HERG protein, distinguishing tumour endometrial cells from normal endometrium.

We then investigated whether HERG protein was expressed also on the plasma membrane of these tumour cells, giving rise to functioning HERG $K^+$ channels. For this reason, patch clamp recordings were performed on primary cell cultures obtained from adenocarcinoma samples, which resulted to be positive at the immunohistochemical analysis. Figure 4 shows typical current traces obtained from these cells. After cell perfusion with high $K^+$ solution, to increase the driving force for inward $K^+$ currents at our test potentials (from +20 to –120 mV), an inward inactivating current was detected in 70–80% of the tested cells (panel A). This current was completely inhibited by the antiarrythmic drug Way 123,398 (panel B). In C is reported the $I_{HERG}$ isolated by substracting the current in presence of Way 123,398 to the native current (see also the inset at higher magnification) (Arcangeli et al, 1995; Sanguinetti et al, 1995; Faravelli et al, 1996). These data indicate that HERG protein, detected in endometrial cancer cells, is indeed expressed on the cell surface of these cells, giving rise to HERG channels with normal electrophysiological features.

The RT-PCR, as well as the immunohistochemical analysis, were then performed on further 29 surgical specimens, characterized by both standard histopathological criteria, and currently used biomolecular markers (estrogen receptors (ER), progesteron receptors (PgR), p53 and bcl-2 proteins). Data relative to these cases are reported in Table 1, showing that ER and PgR were diffusely positive in 17 and 18, respectively, of 18 endometrial adenocarcinoma and in 9 and 11, respectively, of 11 NCE. Bcl-2 protein was diffusely expressed in 16 cases of EC (89%) and in 8 NCE (73%). P53 protein was strongly detected in 13 EC (72%) and in NCE (27%), although present at lower score in 45% of NCE.

 *© 2000 Cancer Research Campaign*

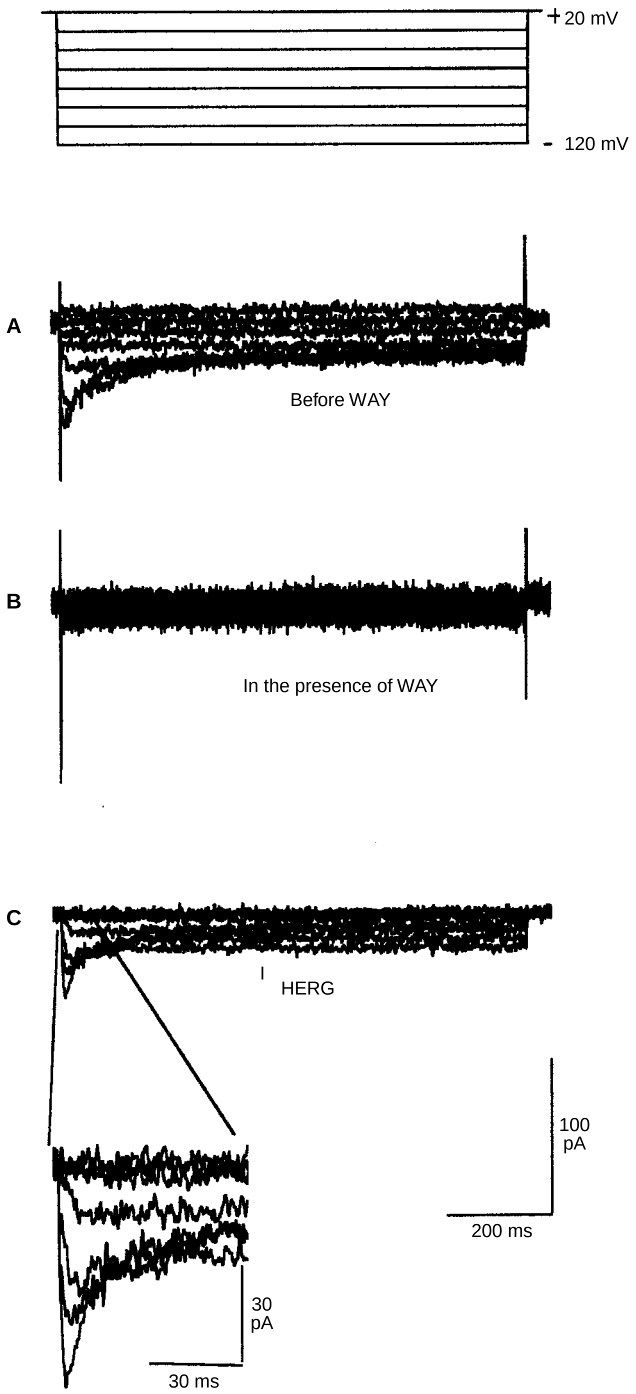

**Figure 4** Electrophysiological detection of HERG currents in endometrial adenocarcinoma cells. Primary cell cultures were obtained from endometrial adenocarcinoma specimens, as reported in Materials and Methods. Patch clamp experiments were performed on single cells after 4–5 days of culture. (**A**) Traces recorded in extracellular solution containing 40 mM [$K^+$], after applying the protocol reported on the top of the figure; (**B**) traces obtained after addition of Way 123 398 (1 μM) in the 40 mM [$K^+$] extracellular solution. (**C**) Isolated HERG currents obtained after substracting the current in the presence of Way 123 398 to the native current. Inset: higher magnification of $I_{HERG}$ reported in **C**. Top: protocol applied to record HERG traces; holding potential was 0 mV.

The parallel analysis of *herg* RNA and HERG protein expression revealed that both are indeed detectable in 67% (12/18) and 82% (14/17), respectively, of cancerous tissues, while are present at lower percentage (18%) (2/11) in NCE. Interestingly, the only NCE cases which resulted to be positive to *herg*/HERG expression were relative to the secretive stage (see Discussion), while all the simple endometrial hyperplasia tested resulted negative.

In tumour cells no significant correlation was found between *herg* RNA and HERG protein with age, histological type, grading, tumour stage or other parameters used (ER, PgR, bcl-2, p53 expression). On the contrary, a significantly higher HERG expression was found between cancerous versus non-cancerous endometrium ($P$ = 0.031 (Chi-square test) and $P$ = 0.021 (Fisher's test)). An even better statistical result was obtained when comparing HERG expression in EC versus endometrial hyperplasia ($P$ = 0.018 (Chi-square test) and $P$ = 0.014 (Fisher's test)).

On the whole, RT-PCR analysis and immunohistochemical experiments gave substantially similar results, revealing that cancerous tissues express both *herg* RNA and HERG protein, the latter being usually detectable in the cytoplasm of adenocarcinoma cells.

### DISCUSSION

$K^+$ channels are evidently involved in the regulation of cell growth and differentiation of normal (Arcangeli et al, 1997), and cancer cells in vitro (Arcangeli et al, 1995, 1998, 1999; Faravelli et al, 1996; Bianchi et al, 1998) in particular, the *eag* family of outward rectifier $K^+$ channels with its members *eag* and *herg*, appears to be deeply involved in cancinogenesis: in fact, the expression of these channels seems to be a selective advantage for tumour cells in vitro (Bianchi et al, 1998; Pardo et al, 1999). However, the presence and role of such $K^+$ channels in primary human tumours are still unexplored. We provide here the first demonstration of the expression of HERG in human primary tumours, showing that the HERG encoding gene, *herg*, and its related protein are expressed in primary endometrial cancers at higher frequency than in non cancerous endometrium. Moreover, a classical $I_{HERG}$ can be recorded on the plasma membrane of tumour cells.

*herg* RNA expression in EC is easily detectable by RT-PCR technique, while the HERG protein could be also detected in EC specimens by using specific anti-HERG antibodies, with an even more selectivity between epithelial tumour cells and glandular normal or hyperplastic tissue (Figure 3 and Table 1). To this purpose, novel polyclonal antibodies were developed, whose immunoreactivity was very similar to that displayed by other widely used anti-HERG antibodies (Figures 2 and 3). In particular, both *herg* RNA and protein were absent in all the simple hyperplasias tested, while they sometimes resulted positive in some normal endometria. It is perhaps worth noting that the only two cases of normal endometrium which turned out positive were both in the secretive stage, a result that, if confirmed in a larger number of cases, could somehow hint that HERG is involved in the regulation of secretion in non-neoplastic endometrial cells, as it is in endocrine cells (Rosati et al, 1998). At this regard, it is worth noting that the RT-PCR analysis, performed on a larger number of NCE, gave the same percentage of positivity, with a selective expression of *herg* RNA in some cases of secretive endometrium (not shown). We want to stress here that, when applied to the same specimens, the two techniques appeared fairly consistent, stressing that no artifact or external contamination occur in the detection of both *herg* RNA and HERG protein; in fact, only two EC cases, indicated as positive by the immunohistochemical technique, were scored as negative with the RT-PCR. In this context, it is important to note that some EC displayed only a focal immunostaining, leading to the

© 2000 Cancer Research Campaign

**Table 1** *herg* RNA and HERG protein expression in primary endometrial carcinomas and normal/hyperplastic endometria.

| Histology | Grade | Stage | ER | PgR | bcl2 | p53 | *herg* | HERG |
|---|---|---|---|---|---|---|---|---|
| 1. S-AC | G1 | II A | 1 | 2 | 1 | 1 | – | + |
| 2. E-AC | G1 | I B | 3 | 3 | 3 | 3 | + | +* |
| 3. E-AC | G1 | I B | 3 | 3 | 2 | 3 | + | + |
| 4. E-AC | G1 | N.D. | 3 | 3 | 3 | 1 | + | + |
| 5. E-AC | G2 | III C | 3 | 2 | 0 | 2 | + | +* |
| 6. MES | G2 | III C | 3 | 3 | 3 | 2 | – | + |
| 7. E-AC | G2 | I C | 3 | 3 | 3 | 3 | + | + |
| 8. E-AC | G2 | I B | 3 | 3 | 3 | 3 | + | ND |
| 9. E-AC | G2 | I C | 3 | 2 | 2 | 3 | + | + |
| 10. E-AC | G2 | I C | 3 | 3 | 3 | 3 | – | – |
| 11. AC-SM | G2 | III A | 3 | 3 | 3 | 2 | + | +* |
| 12. E-AC | G3 | I B | 3 | 3 | 3 | 3 | + | + |
| 13. E-AC | G3 | I B | 3 | 3 | 3 | 0 | – | – |
| 14. E-AC | G3 | I C | 3 | 3 | 3 | 0 | – | – |
| 15. AC-SM | G3 | I C | 3 | 3 | 3 | 3 | + | + |
| 16. AC-SM | G3 | I C | 3 | 3 | 3 | 0 | + | + |
| 17. SP-AC | – | I B | 3 | 2 | 2 | 2 | + | + |
| 18. SP-AC | – | I C | 2 | 2 | 2 | 3 | – | + |
| 19. PE | – | – | 3 | 3 | 2 | 1 | – | – |
| 20. SE | – | – | 0 | 2 | 1 | 0 | – | – |
| 21. SE | – | – | 0 | 2 | 0 | 0 | + | + |
| 22. SE | – | – | 3 | 3 | 3 | 2 | + | + |
| 23. SE | – | – | 2 | 2 | 0 | 0 | – | – |
| 24. SEH | – | – | 3 | 3 | 3 | 2 | – | – |
| 25. SEH | – | – | 3 | 3 | 3 | 1 | – | – |
| 26. SEH | – | – | 3 | 3 | 3 | 0 | – | – |
| 27. SEH | – | – | 3 | 3 | 3 | 3 | – | – |
| 28. SEH | – | – | 3 | 3 | 2 | 0 | – | – |
| 29. SEH | – | – | 3 | 3 | 2 | 0 | – | – |

Histopathological diagnosis, grading and staging were performed using standard procedures, ER, PgR, p53, bcl-2, and HERG protein expression were detected by immunohistochemistry, *herg* mRNA expression was evaluated by RT-PCR. Surgical specimens relative to human EC (cases 1–18), normal endometrium (cases 19–22) and simple hyperplasia without atypia (cases 23–29) were analysed. In case No.4 no staging was evaluated since data have been obtained from an endometrial currettage. S-AC: Secretory adenocarcinoma; E-AC: Endometrioid adenocarcinoma; MES: Mesonephroid or clear cell adenocarcinoma; AC-SM: Adenocarcinoma with squamous metaplasia; SP-AC: Serous-papillary adenocarcinoma; PE: Proliferative endometrium; SE: Secretive endometrium; SEH: Simple endometrial hyperplasia without atypia.
0: ≤ 5%; 1: > 5% ≤ 10%; 2: > 10% ≤ 50%; 3: > 50%. * = Focal expression of HERG protein.

conclusion that the combined use of both techniques could improve the reliability of HERG as a possible tumour marker.

Moreover, the comparison of RT-PCR and immunostaining technique indicates that in normal and neoplastic endometrium *herg* expression correlates with the presence of the translated HERG protein; the latter appears to be particularly abundant in the cytosol and, only rarely, in the nucleus of epithelial cells. This distribution of HERG protein is in agreement with that reported by others in the heart and HERG-transfected cells, using different antibodies (Babij et al, 1998; Zhou et al, 1998). Moreover, the great amount of HERG protein in the cytosol of cancer cells could be explained by the recent discovery that post-translational defects, such as altered N-glycosylation, which often occurs in tumour cells (Taylor-Papadimitriou and Epenetos, 1994), can lead to an intracellular accumulation of HERG protein, with no expression of the protein on the cell surface (Petrecca et al, 1999). In our model the only demonstration of HERG protein expression on the plasma membrane derives from patch clamp recordings, performed on endometrial adenocarcinoma cells; such experiments revealed that a normally functioning $I_{HERG}$ is indeed expressed on the plasma membrane of these cells. This finding further stresses the role of HERG channels in the mechanism of regulation of the resting potential of cancer cells (Arcangeli et al, 1995; Olivotto et al, 1996; Bianchi et al, 1998).

A relevant question to approach in further experiments is also whether HERG expression in primary EC is one of the genotypic alterations accompanying tumour progression, or, as in the case of neuroblastoma (Arcangeli et al, 1998, 1999; Bianchi et al, 1998), is a crucial aspect of the differentiation block that maintains the immature phenotype in transformed cells.

Whatever the merit of these suggestions, our data, showing that *herg* and protein are expressed in primary human tumours with a higher frequency as compared to non-cancerous tissues, could also candidate HERG as a possible molecular marker, capable of discriminating EC from NCE, in particular from simple hyperplasia.

### ACKNOWLEDGEMENTS

The authors are indebted to Dr JM Nerbonne (University of Washington, St Louis, USA) for kindly gifting the polyclonal anti-HERG antibody. This work was supported by grants from Associazione Italiana per la Ricerca sul Cancro (AIRC), Associazione Italiana contro le Leucemie (AIL) Firenze, Consiglio

 © 2000 Cancer Research Campaign

Nazionale delle Ricerche (CNR, Finalized Project ACRO), Ministero dell'Università e della Ricerca Scientifica e Tecnologica (MURST). AC is a fellowship of the Fondazione Italiana per la Ricerca sul Cancro (FIRC).

### REFERENCES

Arcangeli A, Becchetti A, Mannini A, Mugnai G, DeFilippi P, Tarone G, DelBene MR, Barletta E, Wanke E and Olivotto M (1993) Integrin mediated neurite outgrowth in neuroblastoma cells depends on the activation of potassium channels. *J Cell Biol* **122**: 1131–1143

Arcangeli A, Bianchi L, Becchetti A, Faravelli L, Coronnello M, Mini E, Olivotto M and Wanke E (1995) A novel inward-rectifying $K^+$ current with a cell cycle dependence governs the resting potential of mammalian neuroblastoma cells. *J Physiol* (London) **489**: 455–471

Arcangeli A, Rosati B, Cherubini A, Crociani O, Fontana L, Ziller C, Wanke E and Olivotto M (1997) HERG and IRK-like inward rectifier currents are sequentially expressed during neuronal development of neural crest cells and their derivatives. *Eur J Neurosci* **9**: 2596–2604

Arcangeli A, Rosati B, Cherubini A, Crociani O, Fontana L, Passani B, Wanke E and Olivotto M (1998) Long term exposure to Retinoic Acid induces the expression of IRKI channels in HERG channel-endowed neuroblastoma cells. *Biochem Biophys Res Commun* **244**: 706–711

Arcangeli A, Rosati B, Crociani O, Cherubini A, Fontana L, Passani B, Wanke E and Olivotto M (1999) Modulation of HERG current and *herg* gene expression during Retinoic Acid treatment of human neuroblastoma cells: potentiating effects of BDNF. *J Neurobiol* **40**: 214–225

Argentieri TM, Kulik J, DeGennaro LJ, Spinelli W and Colatsky TJ (1998) Inhibition of cardiac delayed rectifier $K^+$ current by overexpression of the Long-QT Syndrome HERG G628S muation in transgenic mice. *Circ Res* **83**: 668–678

Babij P, Askew GR, Nienwenhuijsen B, Su CM, Bridal TR, Jow B, Zhou Z, Gong Q, Epstein ML and Janary CT (1998) HERG channel disfunction in human Long-QT syndrome. *J Biol Chem* **273**: 21061–21066

Backer VV (1996) The molecular biology of endometrial adenocarcinoma, *Clin Obstetr Gynecol* **39**: 707–715

Berchuck A and Boyd J (1995) Molecular basis of endometrial cancer. *Cancer (Phila.)* **76**: 2034–2040

Bianchi L, Wible B, Arcangeli A, Taglialatela M, Morra F, Castaldo P, Crociani O, Rosati B, Faravelli L, Olivotto M and Wanke E (1998) *herg* encodes a $K^+$ current highly conserved in tumors of different histogenesis: a selective advantage for cancer cells? *Cancer Res* **58**: 815–822

Binggeli R and Weinstein RC (1986) Membrane potentials and sodium channels: hypotheses for growth regulation and cancer formation based on changes in sodium channels and gap junctions. *J Theor Biol* **123**: 377–401

Burks RT, Kessis TD, Cho KR and Hedrick L (1994) Microsatellite instability in endometrial carcinoma. *Oncogene* **9**: 1163–1166

Burton JL and Wells M (1998) Recent advances in the histopathology and molecular pathology of carcinoma of the endometrium. *Histopathology* **33**: 297–303

Chatzaki E, Gallagher CJ, Iles RK, Ind TEJ, Nouri AME, Bax CMR and Grudzinskas JG (1994) Characterization of the differential expression of marker antigens by normal and malignant endometrial epithelium. *Br J Cancer* **69**: 1010–1014

Chiesa N, Rosati B, Arcangeli A, Olivotto M and Wanke E (1997) A novel role for HERG $K^+$ channels: spike-frequency adaptation. *J Physiol* **501**: 313–318

Creasman WT (1993) Prognostic significance of hormone receptors in endometrial cancer. *Cancer* **71**: 1467–1470

Faravelli L, Arcangeli A, Olivotto M and Wanke E (1996) A HERG-like $K^+$ channel in rat F-11 DRG cell line: pharmacological identification and biophysical characterization. *J Physiol* (London) **496**: 13–23

Hamill OP, Marty A, Neher E, Sakmann F, Sigworth FJ (1981) Improved patch-clamp techniques for high resolution current recording from cells and cell-free membrane patches. *Pfluegers Arch* **391**: 85–100

Kohler MF, Nishii H, Humphrey PA, Saski H, Marks J, Bast RC, Clarke-Pearson DL, Boyd J and Berchuck A (1993) Mutation of the p53 tumor-suppressor gene is not a feature of endometrial hyperplasia. *Am J Obstet Gynecol* **169**: 690–694

Li X, Xu J and Li M (1997) The human Δ1261 mutation of the HERG potassium channel results in a truncated protein that contains a subunit interaction domain and decreases the channel expression. *J Biol Chem* **272**: 705–708

Maniatis T, Fritsh EF and Sambrook J (1989) *Molecular cloning: a laboratory manual*. Cold Spring Harbor Laboratory, Cold Spring Harbor, New York

Milatovich A, Heerema NA and Palmer CG (1990) Cytogenetic studies of endometrial malignancies. *Cancer Genet Cytogenet* **46**: 41–53

Olivotto M, Arcangeli A, Carlà M and Wanke E (1996) Electric fields at the plasma membrane level: a neglected element in the mechanism of cell signalling. *BioEssays* **18**: 495–504

Pardo LA, Dela Camino D, Sanchez A, Alves F, Brueggemann A, Beckh S and Stuehmer W (1999) Oncogenic potential of EAG $K^+$ channels. *EMBO J* **18**(20): 5540–5547

Petrecca K, Atanasiu R, Akhavan A and Shrier A (1999) N-linked glycosylation sites determine HERG channel surface membrane expression. *J Physiol* (London), **515**: 41–48

Pond AL, Scheve BK, Benedict AT, Petrecca K, Van Wagoner DR, Shrier AS and Nerbonne JM (2000) Expression of distinct ERG proteins in rat, mouse and human heart. *J Biol Chem* **275**: 5997–6006

Porter PL, Gown AM, Kramp SG and Coltrera MD (1992) Widespread p53 overexpression in human malignant tumours: an immunohistochemical study using metharcan-fixed, embedded tissue. *Am J Pathol* **140**: 145–153

Risinger JI, Berchuck A, Kohler MF, Watson P, Lynch HT and Boyd J (1993) Genetic instability of microsatellites in endometrial carcinoma. *Cancer Res* **53**: 5100–5103

Rosati B, Arcangeli A, Cuccuru D, Crociani O, Lecchi M, Olivotto M and Wanke E (1998) Novel properties of ERG $K^+$ channels in pituitary cells. *SFN Annu Meet* **330**: 17 (abstr.)

Sanguinetti MC, Jiang C, Curran ME and Keating MT (1995) A mechanistic link between an inherited and an acquired cardiac arrhythmia: HERG encodes the IKr potassium channel. *Cell* **81**: 299–307

Taskin M, Lallas TA, Barber HRK and Shevchuck MM (1997) bcl-2 and p53 in endometrial adenocarcinoma. *Mod Pathol* **10**: 728–734

Taylor-Papadimitriou J and Epenetos AA (1994) Exploiting altered glycosylation patterns in cancer: progress and challenges in diagnosis and therapy. *Trends Biotechnol* **12**: 227–233

Warmke JW and Ganetzky B (1994) A family of potassium channel genes related to *eag* in *Drosophila* and mammals. *Proc Natl Acad Sci USA* **91**: 3438–3442

Zhou Z, Gong Q, Epstein ML and Janary CT (1998) HERG channel disfunction in human long QT syndrome. *J Biol Chem* **273**: 21061–21066

*© 2000 Cancer Research Campaign*
